# Tobacco smoking and depression: time to move on to a new research paradigm in medicine?

**DOI:** 10.1186/1741-7015-11-138

**Published:** 2013-05-24

**Authors:** Peter de Jonge, Elisabeth Henriette Bos

**Affiliations:** 1 Interdisciplinary Center Psychopathology and Emotion regulation, University Medical Center Groningen, University of Groningen, PO Box 30.001, 9700 RB, Groningen, the Netherlands

**Keywords:** Tobacco smoking, Depression, Heart disease, Cohort study, Nomothetic, Idiographic

## Abstract

A recent paper published in *BMC Cardiovascular Disorders* reported on a study into whether tobacco smoking may serve as a risk factor for depression in patients with heart disease. In the current paper, we discuss several limitations of that study, of which many apply not just to the study itself but to the nomothetic research design that was used. Particularly when bidirectionality between variables is expected, fluctuation in variables over time takes place, and/or inter-individual differences are considerable, a nomothetic research approach does not seem appropriate, and may lead to false conclusions. As an alternative, we describe an idiographic approach in which individuals are followed up over time using many repeated measurements, and from which individual models are estimated. Such intensive time-series studies are not common in medicine, but are well described in the fields of econometrics and meteorology. Combining idiographic research designs with more traditional nomothetic designs may lead to research findings that are not only useful for society but also valid in individuals.

See related research article here http://www.biomedcentral.com/1471-2261/13/35.

## Background

A vast body of literature has reported on the bidirectional association between tobacco smoking and depression. Individuals with depression have a higher risk of smoking, and a decreased chance of being able to quit smoking [1,2]. Smokers in turn have a higher risk of being depressed and a decreased chance of recovery from depression [3]. Both tobacco smoking and depression are associated with serious health consequences, including incident heart disease. Depression and smoking are both considered risk factors for ischemic heart disease [4,5]. In their study published in *BMC Cardiovascular Disorders*, Stafford *et al*. [6] followed up 193 patients with heart disease to investigate the prospective association between smoking and depression and health-related quality of life. The authors concluded that their findings support a role for smoking as an independent predictor of depression in these patients. However, several limitations of the study challenge this conclusion. Many of these limitations are not confined to the Stafford study, but are inherent to the nomothetic research paradigm that dominates medicine.

## Limitations of the study

The Stafford *et al*. study used an observational cohort design to assess patients after hospital admission for a cardiac event. However, we have identified a number of limitations as follows.

1. The extent to which this sample is representative of patients with chronic heart disease or subclinical forms of (unidentified) heart disease is not clear. Moreover, only 193 (37%) of 528 eligible patients were included, further reducing the representativeness.

2. The study power was severely limited by the low prevalence of smokers and of subsequent depression. Conclusions were based on 12 smokers with depression versus 23 non-smokers with depression, numbers that do not warrant multivariate models (the authors used 14 predictor variables).

3. There is the problem of reverse causation. It is likely that the baseline measurement of tobacco smoking would have been affected by a preceding state of depression, and consequently, the observed effects may not be fully attributable to smoking. Stafford *et al*. did try to reduce this effect by statistically controlling for past depression status; however, past depression status (yes/no) is a suboptimal assessment of the confounder. This limitation was further compounded by the fact that smoking was considered only ‘predictive’ of depression at the first assessment, when smoking and depression were measured simultaneously.

4. Suboptimal confounding control also takes place when not all potential confounders, or the wrong confounders, are entered into the multivariate prediction model [7,8]. For example, using only left ventricular ejection fraction to control for the confounding effects of disease severity is unlikely to be sufficient, as the authors also pointed out in their discussion. Likewise, it is unlikely that the models were adequately controlled for confounding by other potential common causes of smoking and depression, such as low self-esteem, emotion-regulation problems, low socioeconomic status, or shared genetic factors.

5. Fluctuations in smoking and depression were not taken into account. Many individuals in the study stopped smoking in the months following hospital admission, but they were counted as smokers throughout the study period.

6. Results obtained at the group level were implicitly generalized to the individual level. From the observation that patients who smoke were more frequently depressed compared to non-smokers, the authors concluded that if an individual stops smoking his or her future depression levels will be lower. This jump from the population to the individual level is often made in epidemiological research, but is only justified if the conditions for ergodicity are met, which include homogeneity (that is, for each subject in the population the same statistical model holds) and stationarity (that is, a process has constant statistical characteristics over time) [9,10]. If these conditions are not met, making this jump from the population to the individual level can lead to false conclusions. Sometimes associations found at the population level are non-existent or even reversed at the individual level [10-12].

## Limitations of the nomothetic approach

Many studies (including our own!) in the medical field have similar limitations to those cited above. It is not our intention to criticize the Stafford *et al*. study specifically, but rather to use it as an illustration of the nomothetic research paradigm, of which the applicability is sometimes overstretched. Some fundamental problems inherent to the nomothetic approach compromise the possibility to draw valid conclusions about individuals [10,13,14].

1. Between-subjects heterogeneity is not well accounted for in nomothetic studies, as data are aggregated over groups of individuals. In fact, the nomothetic approach deals with variability between individuals as if it were error, which is probably one of the reasons why small effect sizes and inconsistent study results are so often reported.

2. Many of the phenomena studied in the medical field show large intra-individual variability. Such fluctuations are not adequately captured in nomothetic studies, because these have only a limited number of assessment waves, separated by large intervals.

3. Third, many medical phenomena are characterized by multiple interactions between several factors, mutually reinforcing effects, and feedback loops. Standard nomothetic study designs and statistical techniques cannot account for such dynamic complexities.

Given these problems, nomothetic studies have little potential to tell us something about causality at an individual level. Instead, they offer what may be called ‘population causality’. Although the generalizability of the results of nomothetic studies to the population may be high, their applicability to specific individuals is low.

## The idiographic approach as an alternative

If we are interested in effects applying to individuals, there is a more feasible alternative approach, referred to as the idiographic approach [9,14,15]. In this approach, power does not result from sample size but from the multitude of repeated measurements. The aim is to explain variance within individuals (intra-individual variation), and the unit of analysis is the individual, which is the most radical way of dealing with heterogeneity and confounding. The multitude of repeated assessments separated by short time lags allows evaluation of the temporal dynamics of the associations of interest, thereby greatly enhancing the possibility of drawing conclusions about causality. Several techniques for the analyses of such high-intensity time series data have been developed in fields such as econometrics and meteorology [16-18], but these techniques have to date hardly penetrated medical research. Capitalizing on the large number of measurements, it is possible to evaluate processes of change over time, bidirectional effects, feedback loops, non-linear effects, and complex dynamic interactions between multiple variables [19,20], effects that are impossible to analyze properly using nomothetic designs. Idiographic studies have the potential to produce results that are of direct clinical relevance to individuals, enabling patient-tailored advice.

## Conclusion and perspectives

The study by Stafford *et al*. is illustrative of some fundamental problems of the current research paradigm, which impede scientific progression. The nomothetic approach is essentially unfit to answer intra-individual questions, because what applies in aggregate is not necessarily informative for what is true for individuals [10]. However, a disadvantage of the idiographic approach, is that results from single individuals do not generalize well to the population at large. Therefore, a combination of nomothetic and idiographic methods, which takes into account both heterogeneity and dynamic complexity, but simultaneously tries to identify similarities and regularities across individuals and time, seems to be the way forward. Some promising steps in this direction have been made in recent years [21-24]. Such an approach should help to identify prototypical patients with specific etiological pathways, and thus arrive at knowledge that is not only useful for society but also valid in individuals.

## Competing interests

Both authors declare no competing interests.

## Authors’ contributions

PDJ initiated the paper, and PDJ and EHB equally contributed to writing the paper. Both authors read and approved the final manuscript.

## Authors’ information

PDJ is a Professor of Psychiatric Epidemiology and Head of the Interdisciplinary Center Psychopathology and Emotion regulation, University Medical Center Groningen, University of Groningen, The Netherlands, who has been involved in several studies on the association between depression and cardiac disease. EB is a post-doctoral researcher at the same institution, who has a background in depression research and time-series analysis.

## Pre-publication history

The pre-publication history for this paper can be accessed here:

http://www.biomedcentral.com/1741-7015/11/138/prepub
